# Nucleophagy—Implications for Microautophagy and Health

**DOI:** 10.3390/ijms21124506

**Published:** 2020-06-24

**Authors:** Florian Bo Otto, Michael Thumm

**Affiliations:** Department of Cellular Biochemistry, University Medical Center Göttingen, Humboldtallee 23, D-37073 Göttingen, Germany; florian.otto@med.uni-goettingen.de

**Keywords:** nucleophagy, piecemeal microautophagy of the nucleus (PMN), nucleus vacuole junction, microautophagy, Atg39, ER-phagy, nuclear pore complexes, endosomal sorting complexes required for transport (ESCRT)

## Abstract

Nucleophagy, the selective subtype of autophagy that targets nuclear material for autophagic degradation, was not only shown to be a model system for the study of selective macroautophagy, but also for elucidating the role of the core autophagic machinery within microautophagy. Nucleophagy also emerged as a system associated with a variety of disease conditions including cancer, neurodegeneration and ageing. Nucleophagic processes are part of natural cell development, but also act as a response to various stress conditions. Upon releasing small portions of nuclear material, micronuclei, the autophagic machinery transfers these micronuclei to the vacuole for subsequent degradation. Despite sharing many cargos and requiring the core autophagic machinery, recent investigations revealed the aspects that set macro- and micronucleophagy apart. Central to the discrepancies found between macro- and micronucleophagy is the nucleus vacuole junction, a large membrane contact site formed between nucleus and vacuole. Exclusion of nuclear pore complexes from the junction and its exclusive degradation by micronucleophagy reveal compositional differences in cargo. Regarding their shared reliance on the core autophagic machinery, micronucleophagy does not involve normal autophagosome biogenesis observed for macronucleophagy, but instead maintains a unique role in overall microautophagy, with the autophagic machinery accumulating at the neck of budding vesicles.

## 1. Introduction

Autophagy is a highly conserved process that facilitates the degradation and recycling of intracellular components and plays a central role in the maintenance of cellular homeostasis. Involved in a wide range of human pathologies, autophagy has garnered attention with respect to novel therapeutics. The autophagic subtype that targets nuclear material is termed nucleophagy. As a principal cellular organelle, regulation of the different nuclear aspects by nucleophagy has emerged as a system with increasing links to ageing, neurodegenerative diseases, as well as cancer [1,2,3].

Nucleophagic processes occur under various conditions. Starvation, rapamycin induced TORC1 inactivation, genotoxic stress, expansion of the nucleus vacuole junction (NVJ), as well as defects in the nuclear envelope and lamina lead to enhanced rates of nucleophagy [3,4,5,6,7]. Detailed investigations towards their initiation showed that macro- and micronucleophagy occur dependent on the Nem1/Spo7–Pah1 axis, a downstream process of TORC1 inactivation [8]. The Nem1/Spo7–Pah1 axis which is part of lipid metabolism, is also involved in other autophagic processes, including endosomal sorting complexes required for transport (ESCRT)-dependent and autophagy independent micro-ER-phagy [9].

Nucleophagy occurs in both a macro- and micronucleophagic manner in the budding yeast *Saccharomyces cerevisiae* [4,5]. Designation of nuclear material for autophagic degradation is mediated by autophagic cargo receptor Atg39, independent of the nucleophagic mode [10]. Nucleophagy involves the formation of micronuclei, small parts of nuclear material that bud off of the main nucleus, which are then degraded by delivery to the vacuole.

Macronucleophagy is a classic example of selective macroautophagy involving the core macroautophagic machinery (hereafter the core autophagic machinery), with the addition of factors conveying selectivity. Macroautophagy is a well investigated process, as are the roles for members of the core autophagic machinery within it. Signaling and biogenesis of the double membraned autophagosome (the vesicle that transports autophagic cargo to the vacuole) require the core autophagic machinery.

While many studies generally focus on the mechanism of (selective) macroautophagy, micronucleophagy in yeast, termed piecemeal microautophagy of the nucleus (PMN), presents a model system for selective microautophagy. Microautophagy is representative of the umbrella terminology that covers processes which involve the direct abstraction of cellular materials into the vacuole/lysosome. The autophagic machinery is required only for certain subtypes [11,12,13]. PMN depends on the autophagic machinery [13,14]. Localisation of the autophagic machinery in PMN is reminiscent of the micropexophagic membrane apparatus (MIPA), required for micropexophagy in *Komagataella phaffii* (former *Pichia pastoris*) [10,15,16]. Its localisation is mirrored by the ESCRT-III complex in micro-ER-phagy [9]. The distinct roles of the autophagic machinery show that while macro- and micronucleophagic processes depend on the core autophagic machinery and can occur under the same conditions, there is an increasing number of features that set them apart.

**Figure 1:**
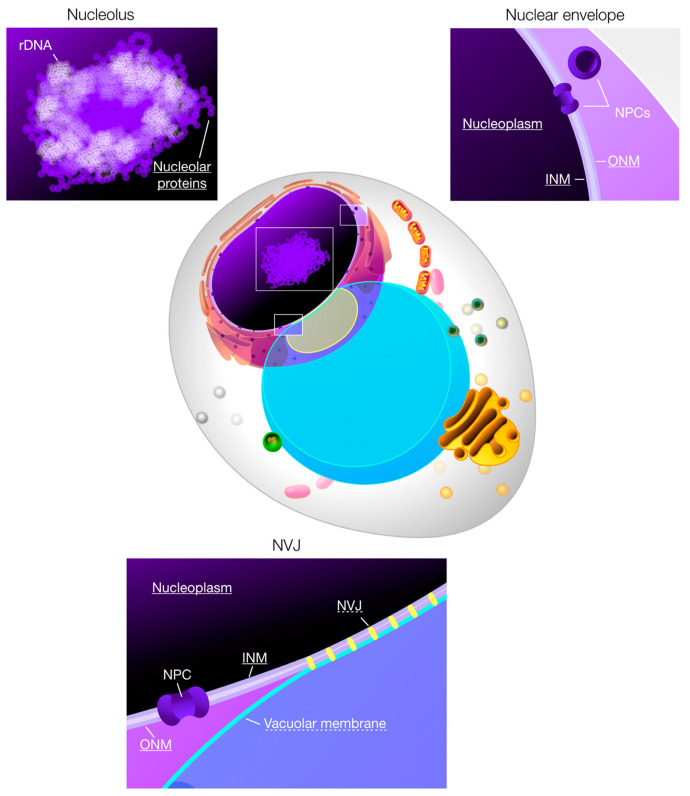
Nucleophagic cargo in context of an *S. cerevisiae* cell. Nucleophagy targets a variety of cargos in yeast. Nuclear compartments relevant for nucleophagy are additionally highlighted in a detailed depiction. Known cargos of nucleophagy are underlined, and cargos exclusive to micronucleophagy show a dashed underline. Nucleophagy results in formation of micronuclei, small portions of nuclear material that bud off of the main nucleus. Inner- and outer nuclear membrane (INM and ONM) are targeted by nucleophagy, excluding nuclear pore complexes (NPCs), which are degraded by a specialised form of selective autophagy. Nucleoplasm, as well as nucleolar proteins are degraded, while excluding ribosomal DNA (rDNA). Piecemeal microautophagy of the nucleus targets these components as well and additionally degrades the tightly clamped nucleus vacuole junction (NVJ), including a portion of vacuolar membrane.

Many cargos are shared between the nucleophagic modes, implicating nucleophagy in regulation of the nuclear envelope and its domains, as well as nucleoplasmic content, including the nucleolus (Figure 1) [5,14]. The nucleus vacuole junction (NVJ), a large compositionally unique membrane contact site between vacuolar membrane and nuclear envelope is an exclusive target of PMN [4,10]. Regulation of the NVJ by PMN has many implications towards its influence on lipid metabolism and both nuclear and vacuolar membrane composition. In contrast, NPCs are excluded from the NVJ and PMN does not result in their degradation [4,17,18,19,20]. Instead, a specialised form of macroautophagy targets NPCs. Nup159 was shown to specifically act as a cargo receptor for macroautophagic degradation of NPCs, which occurs largely independent of Atg39. Similar to Atg39 dependent macronucleophagy, autophagy of NPCs also includes nuclear envelope and a portion of the nucleoplasm [19,20].

Many aspects of the nucleophagic processes are still unknown. The budding process that results in the formation of micronuclei and the nature and composition of a potential microautophagic membrane have yet to be characterised in detail. As the role of the ESCRT machinery in PMN is still unknown, future studies will have to determine its involvement to further understand the details of the microautophagic classes. For many nucleophagic cargos such as the nucleoplasm, it is difficult to determine whether they are actual targets of the autophagic process, or degraded simply by circumstance. To understand the role of nucleophagy, detailed analyses of nucleophagic cargo compositions might distinguish designated from collateral cargos. Regulation of the NVJ by PMN and its implications on lipid metabolism have been hinted at, but direct evidence could substantiate a nucleophagic role in lipid regulation. Ultimately, nucleophagic influence on disease and its utilisation towards uncovering novel approaches to treatment is implied by present data, but still requires further research for meaningful assessments.

## 2. Signaling for Nucleophagy

Nucleophagy was initially shown to occur during nutrient limitation in yeast. TORC1 inactivation by nitrogen starvation as well as rapamycin induces nucleophagy independent of its mode [4,5,10]. Nucleophagic processes begin by formation of micronuclei and TORC1 inactivation leads to the recruitment of the core autophagic machinery to these micronuclei [10,13]. In addition to the core autophagic machinery, nucleophagy specifically requires cargo receptor Atg39 of the nuclear envelope [5,10].

In line with nucleophagic activity upon starvation, the upstream region of *ATG39* contains two stress-response element (STRE) repeats, which upregulate gene expression in stationary phase and nutrient deprivation conditions, supporting a link to nucleophagy [21]. Outer nuclear membrane (ONM) resident Nvj1 is essential for the formation of the NVJ and thereby micronucleophagy. The upstream region of *NVJ1* also contains two STRE repeat regions [22]. Noticeably, overexpression of Nvj1 leads to an enlargement of the NVJ and subsequently higher rates of micronucleophagy [6].

Besides similarities in expressional regulation, localisation of both Atg39 and Nvj1 depends on the Nem1/Spo7–Pah1 axis [8]. The Nem1/Spo7–Pah1 axis is activated as a consequence of TORC1 inactivation and its absence leads to impairment of both macro- and micronucleophagy [8,23]. Previously shown to be required for maintenance of nuclear envelope morphology, the Nem1/Spo7 complex is located in the nuclear envelope [24]. While its influence on nucleophagy is particularly extensive, the complex is also relevant for other modes of autophagy yet does not influence the Cvt-pathway [8]. Pah1 yields diacylglycerol by dephosphorylation of phosphatidic acids. Its capacity for maintenance of the nuclear envelope might be a factor for the Nem1/Spo7–Pah1 axis in modulating the nuclear membrane for micronuclei formation.

Whether a target or a side effect of microautophagic processes, vacuolar membrane degradation is a distinguishing feature of the process. Pah1 mediated diacylglycerol synthesis is required for functionality of overall macroautophagy and micronucleophagy as well as maintenance of nuclear morphology [8]. The Nem1/Spo7–Pah1 axis appears to be relevant beyond nucleophagy, in overall microautophagic context, as core autophagy independent and ESCRT dependent micro-ER-phagy also depends on it [9]. Lipid provision and/or metabolism after microautophagic membrane consumption and involvement in vacuolar domain formation were put forward as potential reasons for its conserved relevance in overall microautophagy [8,9,25,26].

Nucleophagic activity in mammalian cells is linked to oncogenic and genotoxic stress [3,27,28]. While nucleophagy in mammalian cells is initiated by pathological conditions, the Nem1/Spo7–Pah1 axis is conserved from yeast to mammalian cells, as the orthologous CTDNEP1/NEP1R1-lipin complex. The CTDNEP1/NEP1R1-lipin complex is, similar to its counterpart, located in the nuclear envelope [29]. Human CTDNEP1 can functionally complement yeast Nem1 and mutations in CTDNEP1 were discovered in tumor cells [29,30]. Whether nucleophagy is also observed in mammalian cells under physiological conditions will have to be clarified in future studies.

## 3. Mechanism

After signaling, nucleophagic processes begin with the formation of micronuclei. These small portions of nuclear material are then recognised by the autophagic machinery. Their formation has not yet been described in detail. Recruitment of the autophagic machinery to micronuclei most likely follows cargo receptor accumulation [10].

**Figure 2:**
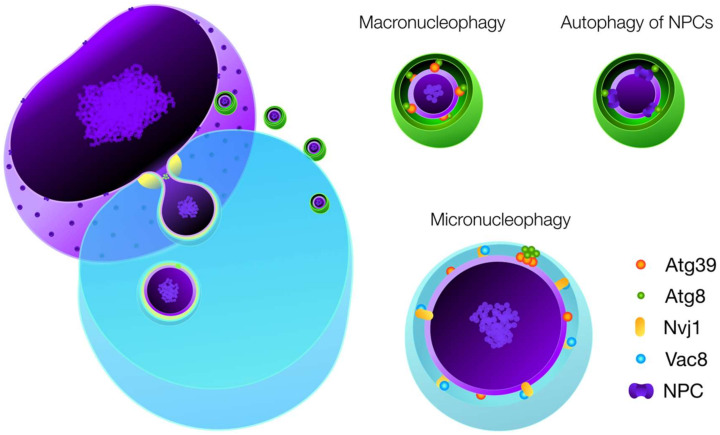
The two principle modes of nucleophagy in *S. cerevisiae*. Macronucleophagy is a selective macroautophagic mode dependent on cargo receptor Atg39. Atg39 interacts with Atg8 in the autophagic membrane to form autophagosomes around micronuclei, which are ultimately transferred to the vacuolar lumen. Autophagy of nuclear pore complexes (NPCs) was shown to be a process separate from macronucleophagy, occurring independent of Atg39, instead recognizing cargo by interaction of Atg8 with Nup159 within the NPC. Micronucleophagy (piecemeal microautophagy of the nucleus) also depends on Atg39. Its larger cargo diameter of ~700 nm for micro- when compared to ~380 nm of macronucleophagic vesicles, restriction to the nuclear vacuolar junction (NVJ) and a punctate Atg8 positive structure between the tips of the vacuolar invagination set it apart from macronucleophagy.

Whereas the autophagosome envelops micronuclei in macronucleophagy the autophagic machinery accumulates at a MIPA-like structure in PMN (Figure 2). Presence of the autophagic machinery is not only highly reminiscent of micropexophagy in *K. phaffii*, but also of ESCRT involvement in micro-ER-phagy [9,31].

### 3.1. Process of Nucleophagy

Nucleophagic processes involve bulging of the nuclear envelope as a first step, followed by budding of the micronucleus. In the ER, membrane bulging is mediated by ER cargo receptor RETREG1. RETREG1 (similar to its *S. cerevisiae* counterpart, Atg40) contains a reticulon homology domain (RHD), which conveys curvature induction required for ER-phagy [32]. In *S. cerevisiae* nucleophagy depends on ONM resident autophagic cargo receptor Atg39 [5,10]. While Atg39 does not contain an RHD, initial bulging of the nuclear envelope for formation of micronuclei could be facilitated by curvature inducing factors that localise to the ONM in tandem with Atg39.

Atg39 interacts with Atg8 via an Atg8-interacting motif (AIM) within its cytosolic N-terminal region. Atg39 additionally interacts with cargo receptor adaptor Atg11 via an Atg11 binding region (11-BR), a feature commonly observed in selective modes of autophagy. Both interactions with Atg8 and with Atg11 are required for macronucleophagy [5]. Deletion of Atg39, its AIM, or 11-BR leads to disruptions of nuclear envelope and the nucleus and entails a decrease in cellular viability [5,33].

Atg8 recruitment follows heightened Atg39 accumulation. Atg39 was shown to accumulate around macronucleophagic cargo prior to Atg8 recruitment. Autophagosome biogenesis follows and the process is finalised by transfer of the vesicle to the vacuolar lumen. Autophagosome transfer of micronuclei coincides with a change in vacuolar membrane composition, presumably a shared feature of overall macroautophagy. In contrast, Atg39 is present surrounding PMN cargo, comparable to its presence in the ONM, and accumulates at a punctate structure between the neck of the vacuolar invagination. The accumulation also coincides with Atg8 and an extended Atg11 presence, displaying a unique role for the autophagic machinery in a microautophagic system [10].

### 3.2. Role of the Autophagic Machinery in Micronucleophagy

The punctate Atg8 positive structure between the tips of the vacuolar invagination in PMN is reminiscent of mechanisms observed in other microautophagic processes. A membrane structure involved in microautophagic subtypes that depend on the core autophagic machinery explains at least in part their requirement, as the components are essential for autophagic membrane biogenesis.

The MIPA is an extended membrane structure that closes the microautophagic structure around aggregated peroxisomes, linking vacuolar sequestering arms [15,16]. Its functionality as a component for closure of the vacuolar membrane surrounding microautophagic cargo, as well as Atg8 presence within the structure are shared between micropexophagy and PMN. Despite these similarities, comparison of the MIPA with the micronucleophagic structure also reveals compositional differences. The MIPA contains *Kp*Atg26, which is required for micropexophagy and formation of the MIPA [31]. In contrast, Atg26 is redundant for the functionality of PMN [13]. Phosphatidylinositol 4-phosphate is a signaling lipid for the MIPA and accumulates within it [31]. Whether the micronucleophagic structure observed in PMN has a distinct lipid composition from autophagosomal membrane has yet to be determined. Whereas PMN cargo bulges into the vacuole, the MIPA links vacuolar sequestering membranes that protrude from the vacuole and envelop cargo. The MIPA presumably seals these sequestering membranes, while PMN is carried out without their formation. The MIPA is also larger than the autophagic structure in PMN. While this could be another distinguishing factor, the MIPA is observed in response to overabundance of peroxisomes, resulting in particularly large microautophagic structures. PMN vesicles on the other hand are formed without overexpression, which is a potential explanation for the smaller, punctate autophagic structure [10,31]. The Atg8 positive, micronucleophagic structure is transferred to the vacuolar lumen alongside its associated cargo vesicle. Its presence is speculated to allow access for vacuolar hydrolases to the cargo vesicle since it presents a site that is not shielded by vacuolar membrane [10].

Core autophagy independent microautophagy typically involves the ESCRT machinery [9]. ESCRT activity resembles microautophagy and is involved in membrane remodeling and severance in various processes including multivesicular endosome formation, NPC quality control, as well as nuclear envelope assembly and repair [34,35,36,37]. Whether the ESCRT machinery is also involved in micronucleophagy has yet to be determined. ESCRT involvement is not exclusive to microautophagy, but is also required for the functionality of macroautophagic processes [38,39,40].

Morphologically and functionally reminiscent of the micronucleophagic process, micro-ER-phagy shares its large vesicle diameter and also displays mechanistic activity at the neck of vacuolar membrane invagination. ESCRT-III component Snf7 is found here as a punctate structure, resembling the situation reported for Atg8 in PMN [9,10]. The ESCRT-III complex is required for membrane scission in the micro-ER-phagy process, rather than for the formation of a microautophagic membrane. Micro-ER-phagy is also a downstream process of the Nem1/Spo7–Pah1 axis [9]. A shared reliance on the Nem1/Spo7–Pah1 suggests requirement for lipid metabolism that preludes microautophagy as a consequence of its membrane consumption, or its potential for vacuolar domain formation.

The ESCRT machinery is also required for both microautophagy and macroautophagy in mammalian cells [39,40,41]. Here, micro-ER-phagy was shown to occur independent of core autophagic proteins required for autophagosome biogenesis (ULK1, ULK2, ATG13, ATG14), but dependent on those required for LC3 lipidation (ATG4B, ATG7, or ATG16L1). This select subset suggests, that micro-ER-phagy in mammalian cells does not require autophagic membrane formation [41].

## 4. Nucleophagic Cargo

The distinction of nucleophagic processes with regard to cargo composition remains difficult, since many cargos are shared between the nucleophagic modes (Table 1). Presumably an effect of substrate composition, macro- and micronucleophagy form vesicles with highly distinct diameters, with micronucleophagy producing significantly larger vesicles than those observed for macroautophagy (Figure 2) [10].

Overall, mammalian cells share nucleophagic cargo types observed in yeast, but additionally include the nuclear lamina, a feature absent from yeast cells. DNA is generally excluded from nucleophagic processes in yeast, but found in micronuclei in mammalian cells [42].

### 4.1. Nuclear Envelope

The nuclear envelope is the confining structure of the nucleus. It is a double membrane, representative of the perinuclear portion of the ER. Required for maintenance of structural integrity and segregation of nucleoplasm and cytoplasm, the nuclear envelope is also required for the spatial organisation of the nucleus. The inner nuclear membrane (INM) forms domains that interact with nuclear components and is associated with the nuclear lamina, a conserved feature of metazoan cells. Micronuclei, precursors to nucleophagic vesicle formation and the vesicles themselves, were shown to include nuclear envelope, independent of the mode. Degradation of nuclear envelope via nucleophagy is linked to regulation of nuclear extent, counteracting disruption of its components and morphology, as well as targeted removal of its subdomains.

As the nuclear envelope harbors cargo receptor Atg39, it is degraded by macro- and micronucleophagy [10]. Nucleophagy thereby targets both ONM as well as INM, resulting in double ringed structures detected within corresponding autophagosomes [4,5]. Both nuclear membranes are also core aspects of the NVJ, clamped together by Nvj1, and are degraded alongside vacuolar membrane by micronucleophagy [43].

Present in mammalian cells but not in yeast, the nuclear lamina is associated with the nuclear envelope. It is a cytoskeleton meshwork providing mechanical stability and organisation of chromatin, located at the INM. The lamina contains multiple lamins and associated proteins. Downregulation of nuclear lamina proteins occurs upon senescence within days, but their long-lived structure suggests active removal of present proteins by a targeted turnover process [3]. LC3 interacts with lamin A/C, emerin, as well as lamin B1 of the nuclear lamina [27,44]. As opposed to Atg8, which interacts with cargo receptors on the cytosolic face of the nuclear envelope for nucleophagy, LC3 is conditionally localised in the nucleus and directly interacts with lamins of the nuclear lamina. Nuclear localisation of LC3 was shown previously and is regulated by de-acetylation upon starvation, which results in its redistribution to the cytosol for the initiation of autophagy [45]. Interaction of LC3 with the nuclear lamina and nucleophagy of lamina components were shown to affect nuclear morphology and cellular viability [7,44].

LC3 interacts with lamin B1 at lamin-associated chromatin domains (LADs) [44]. LADs are regions of chromatin contact with the lamina. Autophagy was shown to degrade chromatin fragments that bud off of the main nucleus [46]. Nuclear envelope sites targeted by nucleophagy lack nuclear envelope protein SUN1, which mediates nuclear anchorage, suggesting a distinct composition of designated cargo sites [47]. Lamin B1 degradation does not occur under nutritional stress, but instead upon cellular senescence, induced by oncogenic and genotoxic conditions [39]. Lamin A/C and emerin deficient cells show LC3 positive autophagosomes tethered to the nuclear envelope. Inhibition of autophagy was shown to result in disturbance of nuclear morphology and compromises cellular vitality, a potential result of lacking means towards removal of damaged nuclear content [7].

### 4.2. NPCs

NPCs allow bidirectional transport of macromolecules from- and to the nucleus and are distributed over the nuclear envelope. Autophagic degradation of NPCs allows bulk degradation of these macromolecular structures and is a process separate from macronucleophagy, as it does not require Atg39 [19,20]. As a consequence of their absence from the NVJ, NPCs are not degraded by PMN [14]. Autophagy of NPCs is a countermeasure for disrupted complexes and regulates the overall quantity of NPCs within the nuclear envelope.

Nuclear protrusions were reported to contain NPCs and macroautophagy of NPCs was shown to enable NPC turnover either upon induction by starvation or NPC disruption [19,20,48]. Analogous to Atg39 located in the ONM, NPC component and Nup159 also contains a functional cytoplasmic AIM. As with macro- and micronucleophagy, and in line with its selective modality, macroautophagy of NPCs also additionally requires presence of- and interaction with Atg11. Autophagy of NPCs degrades multiple NPCs alongside nuclear envelope and a portion of the nucleoplasm [19]. Autophagic degradation of NPCs was observed under nitrogen starvation conditions and also upon introducing mutations that cause NPC clustering, suggesting regulatory in addition to bulk protein degradation observed as a result of starvation [19,20].

In mammalian cells, nuclear envelope protrusions associated with autophagic degradation were shown to contain NPCs [42]. Impairment of NPC assembly, as well as complex disruption, is linked to ageing, neurodegeneration and cancer [49,50,51].

### 4.3. Nucleolus

The nucleolus is the largest sub-nuclear structure. It is a site of ribosome biogenesis and assembles around ribosomal gene (rDNA) arrays. Beyond its relevance for ribosome biogenesis, the nucleolus continues to emerge as a hub for regulation of many cellular processes, including the regulation of gene expression, nuclear organisation and maintaining cellular homeostasis. Nucleolar components are degraded by nucleophagy and rDNA is retained by a coordinated separation mechanism. Pathological increase of nucleolar size has been observed in various diseases and its continuous expansion has been linked to age. Regulation of the nucleolar components is a requirement for maintenance of cellular viability and size reduction was correlated with a significant expansion of cell lifespan [1,52,53,54].

Nucleophagy results in the selective degradation of parts of the nucleolus. Fibrillarin ortholog and Ribi (ribosome biogenesis/maturation) protein Nop1 was shown to be a prominent cargo of both macro- and micronucleophagy [5,14]. TORC1 inactivation results in reduction of nucleolar size and condensation of rDNA in yeast and mammalian cells [55,56]. Of note, overabundance of Nop1 is toxic and while it is degraded under native conditions by nucleophagy, actively overexpressed Nop1 is additionally targeted by proteasomal degradation via the CUE domain-containing Def1 [57].

Nucleolar size has been shown to increase relative to cellular age in *Caenorhabtitis elegans* and mammalian culture of Hutchinson–Gilford progeria syndrome (HGPS) derived cells [1,52]. HGPS cells express progerin, a mutant form of lamin A. Depletion of lamin A and expression of progerin both result in nucleolar expansion [1]. Rapamycin induces mutant protein clearance in HGPS cells and counteracts its cellular phenotype [58]. Lifespan expanding *C. elegans* mutants generally maintain smaller nucleoli and in analogy to Nop1, fibrillarin expression expands nucleolar size and results in progeria. NCL-1, homologue of tumour suppressor TRIM2/Brat inhibits production of fibrillarin and promotes lifespan, whereas its deletion causes nucleolar expansion [53,54].

### 4.4. Nucleic Acids

DNA, specifically rDNA, is separated from the nucleolus and condensed in the initial stages of nucleophagy, allowing for degradation of nucleolar proteins while retaining rDNA. rDNA exclusion from nucleophagic cargo is mediated by two mechanisms, the CLIP cohibin system that retracts rDNA to the INM and condensation of rDNA [55,59,60,61]. DNA damage induces autophagy in yeast by a process termed genotoxin-induced targeted autophagy (GTA) and defective autophagy inhibits DNA repair. GTA is a selective autophagic mode that requires Atg11, but does not require any of the known cargo receptors including Atg39. Genotoxicity and double-strand break initiate GTA independent from TORC1 inactivation, instead requiring an alternate pathway that includes Mec1/ATR, Tel1/ATM, and Rad53/CHEK2, which are largely dispensable for TORC1 mediated autophagy [62].

In contrast, DNA degradation and nucleophagy are interlinked in mammalian cells. LC3 positive micronuclei contain DNA alongside DNA damage marker γH2AX [63]. DNA damage prompts nucleophagy, resulting in degradation of lamin A/C alongside leaked DNA [28]. DNA degradation alongside chromatin was shown to be carried out by autophagy in cancer cells. Here, arginine starvation was used to induce DNA as well as chromatin leakage which leads to subsequent degradation by autophagy, specifically killing tumor cells [42].

### 4.5. NVJ

The NVJ is a uniquely composed membrane contact site between nucleus and vacuole [64,65]. A site for lipid metabolic activity and membrane lipid exchange, it is a prerequisite for and at the same time exclusively degraded by PMN [4]. Linking nucleophagic processes and lipid metabolism also explains identification of Atg39 as a factor for both [5,33].

Formation of the NVJ facilitated by interaction of Nvj1 in the nuclear envelope and Vac8 in the vacuolar membrane [18]. The competitive interaction with Atg13 and Nvj1 differentially regulates the Cvt and PMN pathways [66,67]. Vac8 is required for many micro- and macroautophagic modes [68,69,70,71,72]. The unique composition of the NVJ is at least in part due to Nvj1, which recruits various other proteins. Osh1, a functional homologue to the oxysterol-binding proteins (OSBPs) within the mammalian system, was shown to transport sterols via its OSBP-related domain (ORD) presumably by exchange with PI4P [73]. The NVJ was shown to be a sterol enriched membrane domain, which is typically liquid ordered [14,25]. The essential enoyl-CoA reductase Tsc13 is also recruited to the NVJ in a Nvj1 dependent manner and is presumed to catalyse synthesis of very long chain fatty acids. Attenuation of its fatty acid elongation activity was shown to result in a significant decrease of PMN vesicle diameter, implying a compositional dependence on vesicle size [74].

Mdm1 additionally tethers the NVJ and its overexpression results in hypertethering of ONM to the vacuole, creating contact sites independent of Nvj1. Mdm1 is a substrate of PMN, yet dispensable for its functionality [75,76]. It is highly conserved in mammals as four sorting nexins (Snx13, Snx14, Snx19, Snx25). Snx14 mutations are implicated in neurological disease (autosomal-recessive cerebellar ataxia) and introducing disease analogous mutations in Mdm1 impairs its tethering functionality [75,77]. Lipid droplets accumulate at the edges of the NVJ and Mdm1 is involved in their recruitment and synthesis [76,78]. Recruitment of lipid droplets to the NVJ is speculated to be a mechanism for the removal of toxic lipids, such as free fatty acids, from the ER, which could subsequently be degraded by microlipophagy [79].

Noticeably Atg39 was also identified as ER-stress-induced microlipophagy gene 1 (Esm1), linked to microlipophagy and ESCRT machinery, required for adaption to lipid imbalance after lipid- and ER-stress [33]. Lipid homeostasis is influenced by the NVJ and its autophagy dependent regulation via Atg39-dependent micronucleophagy. Due to association of the aminoterminus of Nvj1 with the INM within the NVJ, the nuclear envelope shows a distance of approximately 8.7 nm from ONM to INM, whereas outside it shows a distance of approximately 18.7 nm [43]. The tight clamping might prevent simple dissociation of the NVJ. Instead, micronucleophagy as a regulatory process of the NVJ provides a fast and comprehensive degradation mechanism [10,74].

## 5. Outlook

Nucleophagy has recently emerged as a highly investigated topic, yet many of its aspects remain to be elucidated. While it is a selective mode of autophagy, and reference with other selective autophagic processes furthers the understanding of nucleophagy, many comparisons have limited relevance for the process due to the fact that nucleophagy targets only a select portion of the organelle, adding an additional level of complexity to its selective modality.

Involvement of reticulons, as observed for autophagic turnover of cortical ER via Atg40 for initial membrane bulging, could also be an intriguing mechanism for labelling sub-organellar sites for nucleophagic turnover and requires further investigation.

Identification of a structure resembling the MIPA needs to be addressed in compositional reference with the situation observed in *K. phaffii*. Analysis of the microautophagic structure will likely allow for a better understanding of the role the core autophagic machinery assumes in microautophagy.

Parallels regarding ESCRT involvement in micro-ER-phagy should be addressed by investigation of ESCRT components in relation to nucleophagy and whether their absence has an impact on nucleophagic cargo turnover.

A more comprehensive understanding of nucleophagic cargo with regard to underlying coordination of its components such as DNA, but also protein content and thereby a possible differentiation of the nucleoplasmic portion would greatly benefit the understanding of nucleophagy. Compositional analysis of micronuclei and a connection of NVJ, micronucleophagy and lipid metabolism will enable a more comprehensive insight into the relevance of nucleophagic processes regarding cellular homeostasis and its associated disease conditions.

## Figures and Tables

**Table 1:** Known nuclear cargos and their associated autophagic modes described in *S. cerevisiae*.

Cargo	Autophagic Mode	Specifications	Reported Cargo Components	Reference
Nuclear envelope	Macronucleophagy, micronucleophagy, autophagy of NPCs	Macro- and micronucleophagy harbor Atg39	Sec63, Hmg1, Src1, Atg39	[4,5]
NPCs	Autophagy of NPCs, macronucleophagy (partially)	Harbor Nup159, the cargo receptor required for autophagy of NPCs	Many NPC sub-components including Nup159	[19,20]
NVJ	Micronucleophagy	Requires Nvj1 and Vac8 for formation	Nvj1, Vac8, Osh1	[4,6,13]
Nucleoplasm	Macronucleophagy, micronucleophagy, autophagy of NPCs	Fusion of the Nab2 localisation signal with fluorescent proteins	Nuclear localisation signal of Nab2	[10,13,19]
Nucleolus	Macronucleophagy, micronucleophagy	rDNA and nucleolar proteins are separated prior to nucleophagy	Nop1	[5,14]

## Abbreviations

11-BR	Atg11-binding region
CLIP	chromosome linkage INM protein
ESCRT	endosomal sorting complexes required for transport
GTA	genotoxin-induced targeted autophagy
INM	inner nuclear membrane
LAD	lamin-associated chromatin domains
MIPA	micropexophagy-specific membrane apparatus
NPC	nuclear pore complex
NVJ	nucleus vacuole junction
OSBP	oxysterol-binding protein
ORD PMN RHD	OSBP-related domain piecemeal microautophagy of the nucleus reticulon homology domain
STRE	stress-response element
